# Calcium Carbonate Mineralization of Microalgae

**DOI:** 10.3390/biomimetics7040140

**Published:** 2022-09-23

**Authors:** Panagiota D. Natsi, Petros G. Koutsoukos

**Affiliations:** 1 Institute of Chemical Engineering Sciences, FORTH/ICE-HT, 26500 Patras, Greece; 2 Laboratory of Inorganic & Analytical Chemistry, Department of Chemical Engineering, University of Patras, 26500 Patras, Greece

**Keywords:** calcium carbonate, mineralization, microalgae, precipitation, rate of

## Abstract

Biological substrates catalyze the nucleation and growth of sparingly soluble salts however, the underlying mechanism is largely unknown. In the present study, the growth of calcium carbonate (CaCO_3_), on *Acutodesmus obliquus* (AO) microalgae was investigated. The test microalgae favored the growth of CaCO_3_ from solutions supersaturated with respect to calcite (7.94 < SR_calcite_ < 104.71). The precipitation of calcite on AO was not preceded by measurable induction times, and the rates of calcite crystal growth were higher for higher microalgae cell concentrations. The presence of the microalgae cultivation medium and illumination of the supersaturated solutions accelerated the precipitation of CaCO_3_, increasing the rate by 75% in comparison with the respective value in its absence. AO cultures, air dried at 25 °C yielded higher precipitation rates, in comparison with the respective rates in the presence of active AO cultures. At 70 °C, nucleation and growth were suppressed, due to the destruction of the molecular structure of the microalgae. The CaCO_3_ precipitation rates on calcite precipitated on air-dried AO culture, were doubled in comparison with the respective rates obtained with the respective quantities of each component of the composite substrate.

## 1. Introduction

Membrane fouling in water and wastewater treatment, industrial equipment, and marine equipment [1,2] is a common and persistent problem. Bacteria and microorganisms may form thin biofilm layers on the surfaces they are in contact. Biofilms consist of aggregates of microbial cells adhering on surfaces, encapsulated in polysaccharide matrices. The nature and extent of deposit formation varies according to the local environment, which determines their structural characteristics [3].

The formation of deposits of biological origin (biofouling) is often associated with deposits of insoluble inorganic salts. The relationship between the organic matrix and the inorganic precipitates presents a challenge in modern research. Biofouling, refers to the accumulation of undesired biological material, originating from micro and macro-organisms, on surfaces in the form of biofilms. Composite deposits, consisting of bio-deposits associated with crystalline mineral salts form even though there is no crystallographic affinity [4].

The main biofouling mechanism starts with the fluid flow-driven transport of microorganisms. Next, the microorganisms attach with electrostatic interactions to surfaces, forming biofilms [5,6,7]. The formation of biofilms is followed by the growth of microorganism cells on the surface, through the consumption of nutrients present in the solution. The extracellular polymeric substances (EPS) are instrumental in the formation of biofilm. They are mainly secretions of microorganisms with high molecular weight, composed of polysaccharides, proteins, nucleic acids, and lipids [8]. EPS provides for the interconnection of microorganisms, resulting in the formation of a three-dimensional matrix. As a result, EPS plays an important role in the physicochemical characteristics of the microorganism aggregates: mass transfer, surface characteristics, and their ability to be attached to surfaces [9,10].

Contact of aquatic systems with atmospheric carbon dioxide and the presence of photosynthetic microorganisms, in combination with relatively high levels of calcium concentrations often results in the formation of calcium carbonate (CaCO_3_) deposits associated with photosynthetic microorganisms. The formation of calcite, the thermodynamically most stable polymorph of CaCO_3_, in supersaturated solutions, caused by the presence of microbial cells, biosynthetic products, or products of their metabolic activity, is known as microbially induced calcite precipitation (MCIP) [11]. Bicarbonate (HCO_3_^−^) and calcium ions, produced through metabolic activity do contribute to the formation of calcite in the microenvironment of the microbial cells [12]. Despite the fact, that the formation of calcite, has been reported only in association with extracellular components, several studies suggested that intracellular CaCO_3_ precipitation takes place in cyanobacteria as well [13]. Head et al. (2000) reported that calcite may precipitate intracellularly [14]. More recently, Xu et al. (2019) reported calcite and aragonite precipitation, via cell membrane lysis, induced in cyanobacteria [15].

Cell surfaces are very efficient for carbonate nucleation and crystal growth. Cell walls with negatively charged functional groups, such as carboxyl, phosphate, and amino groups, are capable of adsorbing metal ions [10]. For example, the cell wall of *Bacillus subtilis* can bind a significant amount of Mg^2+^, Fe^3+^, Cu^2+^, Na^+^, K^+^, Mn^2+^, Zn^2+^, Ca^2+^, Au^3+^, and Ni^2+^ ions [16]. Moreover, metabolic processes during the growth of microorganisms, play a crucial role in the formation of CaCO_3_ [17]. Depending on the environment, in which MCIP takes place, a corresponding metabolic process is likely to be the determining factor. The presence of *Acutodesmus obliquus* (AO) in freshwater resulted in the production of rather high lipids concentrations increasing respectively the concentration of carboxyl groups [18]. Relatively little however is known concerning the mechanism of the mineral phase formation in the presence of photosynthetic microalgae like AO. The presence of AO in solutions supersaturated with respect to calcium carbonate may provide additional active sites for nucleation and growth. In the present study we investigated the mechanism of the formation of CaCO_3_ in the presence of AO from measurements of the kinetics of precipitation of calcite in the presence of AO in supersaturated solutions. The most important factors involved with the heterogeneous nucleation and crystal growth of calcite in the presence of these biological substrates were calculated from the kinetics data. The stability domain of the CaCO_3_ supersaturated solutions was investigated at free drift conditions. More precise measurements of the rates of precipitation were done at constant supersaturation, in which the thermodynamic driving force for the precipitation of CaCO_3_ was kept constant.

## 2. Materials and Methods

### 2.1. Acutodesmus obliquus (AO) Culture Development

**Figure 1:**
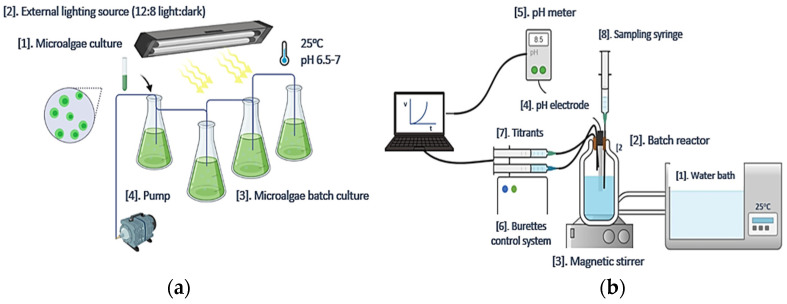
Experimental setup for (**a**) AO culture preparation and (**b**) Experimental setup for the mineralization experiments.

The freshwater microalgae AO was selected for the experiments, as a typical representative of photosynthetic microalgae. These microalgae strains were identified in the water bodies contaminated with industrial effluents [19]. The AO culture was obtained from the Culture Collection of Algae at Göttingen University (*Sammlung von Algenkulturen der Universität Göttingen*, SAG) (Strain number 276-1). Cultures of this green photosynthetic microalgae were grown in a specific culture medium. Following the preparation of the Basal Bolds culture Medium (BBM) [20], the microalgae population was grown in 250 mL conical flasks (batch cultures) (Figure 1a). The composition of the culture medium is shown in Table 1. The supersaturated solutions, upon inoculation with AO cultures, were enriched with nutrient components, the final concentration of which is also shown in Table 1. Air was continuously pumped through the batch cultures, while the illumination conditions selected favored the growth of microorganisms. Cold white 3500 lx lamps were used. Cultures were grown at 25 ± 2 °C and the pH was adjusted to 6.5 with standard solution 0.1 M NaOH.

Four cultures were grown for comparison reasons and measurement of chlorophyll concentration spectrophotometrically.

### 2.2. Precipitation of CaCO_3_ in Supersaturated Solutions

Calcium carbonate (CaCO_3_) precipitation was studied in solutions, supersaturated with respect to all CaCO_3_ anhydrous polymorphs. The experimental setup is shown in Figure 1b. The supersaturated solutions were prepared in a double-walled glass (Pyrex^®^) reactor, thermostated at 25.0 ± 0.2 °C. by the circulation of water of a thermostat through the walls of the reactor. Sodium bicarbonate (NaHCO_3_) and sodium carbonate (Na_2_CO_3_) stock solutions were prepared from the respective crystalline solids (Merck, Puriss.) by dissolution in triply distilled water. The solutions were filtered through 0.2 μm membrane filters (Millipore, Bedford, MA, USA) and were used without further standardization. Fresh solutions were prepared for each experiment. Calcium chloride stock solutions were prepared from calcium chloride dihydrate salt (Merck, Kenilworth, NJ, USA, Puriss.). The stock solutions were standardized with titration with 0.1 M Ethylenediaminetetraacetic acid (EDTA), standard solution, pH 10 with NH_3_/NH_4_^+^ buffer solution, with murexide indicator, and by atomic absorption spectrometry (AAS, AAnalyst 300, Perkin Elmer, Norwalk, CT, USA). The supersaturated solutions were prepared by mixing equal volumes of calcium chloride and sodium bicarbonate stock solutions. The solution’s pH was adjusted to 8.5 with 0.1 M NaOH standard solution, standardized with potassium hydrogen phthalate. Next, the supersaturated solutions were inoculated with known volumes of suspensions of AO cultures so that the number of AO cells introduced in the reactor was known. Alternatively, the supersaturated solutions were inoculated with weighted amounts of solids. Dry AO culture (dried at 25 or 70 °C), CaCO_3_ crystals on which microalgae was grown, and CaCO_3_ which was precipitated on air dried AO culture. Calcite was used as the reference system since in this case there is no nucleation energy barrier and crystal growth started immediately on the calcite crystals upon their suspension in the supersaturated solutions. For this reason, calcite crystals were prepared by precipitation and aging (the specific surface area was 0.33 m^−2^·g^−1^). Calcite precipitation was accompanied by a decrease in the pH value of the supersaturated solutions, because of the release of protons:

(1) $${\mathrm{Ca}}^{2+}\left(\mathrm{aq}\right)+x{\mathrm{HCO}}_{3}^{-}\left(\mathrm{aq}\right)\to {\mathrm{CaCO}}_{3}\left(s\right)+xH^{+}\left(\mathrm{aq}\right)$$

Two different methods were used. The first, known as the free-drift method, mainly aimed to measure the metastable zone width (MZW) for the CaCO_3_ solutions depending on the inoculating material. In this method, the induction time for the onset of precipitation was measured from the time elapsed between the preparation of the supersaturated solutions till the beginning of the solution’s pH drop. The second methodology aimed at accurately measuring the rates of precipitation since accurate measurements are needed for the investigation of the mechanism.

#### 2.2.1. Precipitation of CaCO_3_ from Supersaturated Solutions: Free-Drift Method

Once precipitation in the supersaturated solutions started, the activities of all ions in the supersaturated solutions decreased (Equation (1)). The variation of key parameters was monitored. Solution pH was recorded as a function of time. Samples were withdrawn (with the higher frequency following the start of precipitation and lower closer to completion as indicated by the pH changes, monitored in real-time, and recorded), filtered through membrane filters (0.2 μm Millipore) and the filtrates were acidified, and then analyzed for calcium with atomic absorption spectrometry (AAS, Perkin Elmer AAnalyst 300). The decrease of all parameters continued until no further macroscopic change was observed. At the end of the precipitation process, the suspension in the reactor was filtered and the solid on the filters was characterized by powder X-ray diffraction (XRD, Siemens D-500, Frankfurt, Germany) and scanning electron microscopy (SEM, Leo Supra with Bruker XS EDX microanalysis unit, Zeiss, Oberkochen, Germany). Infra-Red spectra (Attenuated Total reflection—Fourier Transformed Infra-Red, ATR-FTIR) of the solid were recorded in Nicolet 6700 spectrometer (Thermo Scientific, Waltham, MA, USA) equipped with a diffuse reflectance (DRIFTS) cell (Spectra Tech, Thermo Scientific, Waltham, MA, USA).

#### 2.2.2. Precipitation of CaCO_3_ at Constant Supersaturation

Solution pH dropped by as little as 0.01 units, triggering the addition of titrant solutions from two mechanically coupled burettes, mounted on a motorized syringe pump. The two burettes contained calcium chloride and carbonate solutions at concentrations calculated so that they had the stoichiometry of Calcium:Carbonate = 1:1, as in the precipitating solid. As a result, the activities of all ions in the solutions and hence, the respective supersaturation was kept constant. The volume of the titrants added, as a function of time, needed to keep the supersaturation constant, was recorded and from the corresponding graphs, the deposition rates of CaCO_3_ were calculated. sampling was done randomly during precipitation and at the end of the crystal growth, as described in Section 2.2.1.

## 3. Results

### 3.1. Measurement of Growth Rate of Microalgae AO

**Figure 2:**
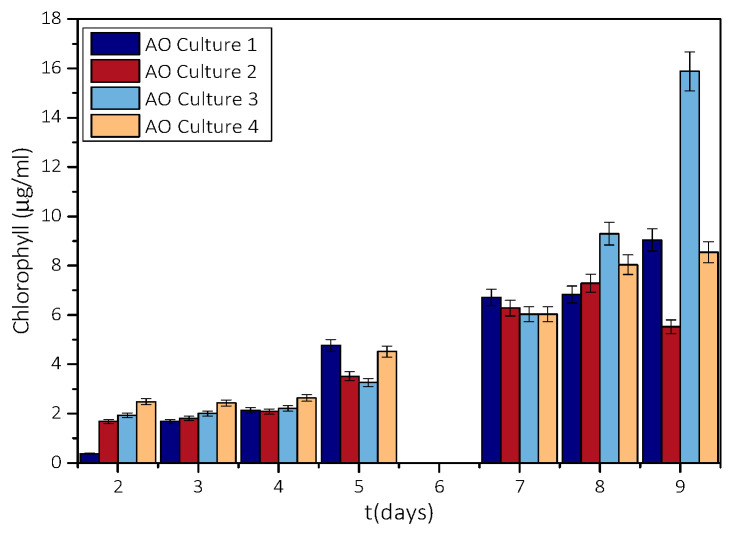
Variation of total chlorophyll with the time of different AO cultures.

Microalgae cultures were grown in 250 mL conical flasks under appropriate illumination and aeration conditions to achieve an optimum growth rate [20]. Samples were withdrawn from the culture suspensions, at regular intervals, and the optical density of the aqueous phase, separated from the solids by filtration, was measured at 650 nm. The optical density is a measure of the concentration of (total) chlorophyll produced during algal growth. AO growth rate was calculated from the change in total chlorophyll concentration in the aqueous phase of the culture. The change in chlorophyll concentration is shown in the plot in Figure 2.

As may be seen, chlorophyll in the aqueous phase of the culture increased with time exponentially. From the measurements of chlorophyll concentration, the growth rate of the microalgae was calculated from the slope of the logarithm of the chlorophyll produced as a function of time, equal to 2 μg·h^−1^. The rates measured were lower (about three orders of magnitude) in comparison with the literature report [21], but it was sufficient to provide sufficient biomass for use as a substrate in the nucleation and crystal growth part of the study.

**Figure 3:**
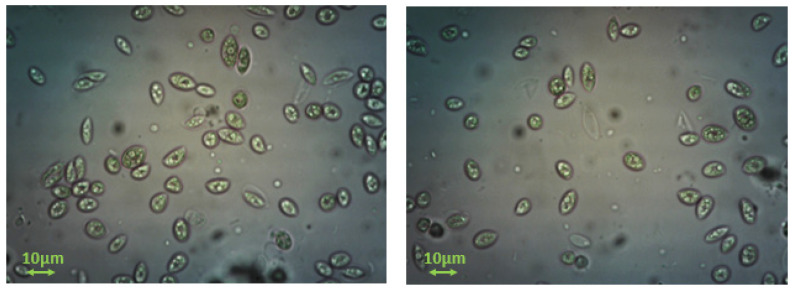
Optical microscope photographs of two different AO cultures (×400) show the same morphological characteristics.

For the calculation of the rate of growth of AO, the cell population was measured by counting the number of microalgae cells, using a hemοcytometer. During the measurements, sampling was done directly from the culture. A few μL was placed on the plate of the hemοcytometer (Neubauer plate), covered with a glass surface, to immobilize the cells. Using an optical microscope (×40) and a camera, photographs were taken (Figure 3) for about a week, during which the microalgae cell population was counted using ImageJ^®^ software [22]. The morphological characteristics of different cultures, grown in different batches were identical.

**Figure 4:**
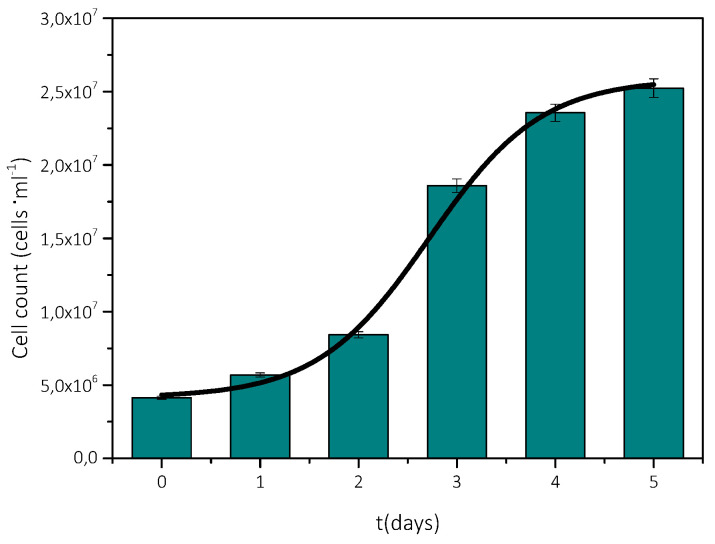
AO culture cell density as a function of time.

From the population measurements, the concentration (number of cells per mL) was calculated, and the results are shown in Figure 4. As may be seen, the population increase was exponential during the first three days. Past the 5th day, it showed a stabilization trend, in agreement with the optical density measurements. Moreover, the transition of the microorganism to the stationary phase of its growth was confirmed.

**Figure 5:**
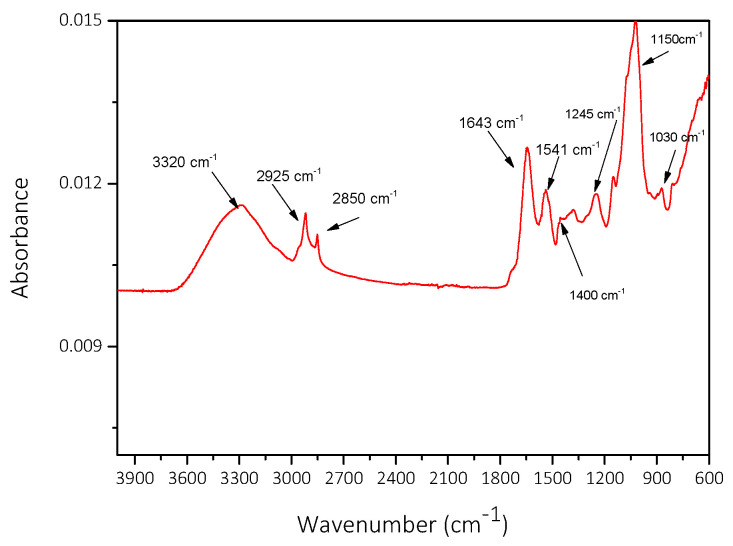
ATR-FTIR spectrum of *Acutodesmus obliquus* dried at 25 °C.

The ATR-FTIR spectra (A) of dried *Acutodesmus obliquus* are shown in Figure 5. The absorbance band between 3500 and 3000 cm^−1^ was typically related to hydroxyl groups. The two intensive bands on 2850 and 2925 cm^−1^ corresponded to CH_2_ symmetric and asymmetric open stretch vibrations. The bands from 1030 to 1541 cm^−1^ correspond to a methyl group stretch assigned to O-CH_3_. The band around 1643 cm^−1^ corresponded to asymmetric vibrations assigned to the -CH_3_ group [23,24].

The infrared spectra of the dried AO culture were in good agreement with literature reports of the ATR-FTIR spectra of chlorophyll and Chlorella vulgaris microalgae [23]. It has been shown that lipid membranes have a substantial calcium-binding capacity, with several types of binding sites present [25]. The significant presence of lipids in AO cultures is therefore expected to promote nucleation and growth of CaCO_3_ as calcium binding may favor the development of active sites for CaCO_3_ growth on the lipid membranes.

**Figure 6:**
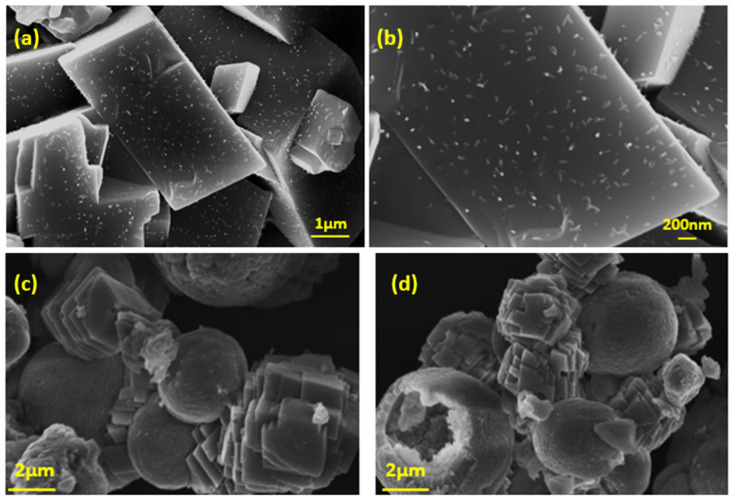
Scanning electron microscope (SEM) photographs (**a**), (**b**) of microalgae cells grown and/or attached to flat faces of rhombohedral calcite crystals (**c**), (**d**) CaCO_3_ spontaneously precipitated on dried AO culture suspended in supersaturated solutions.

AO microalgae cultures were grown on calcite, the thermodynamically most stable CaCO_3_ polymorph. Specifically, calcite powder was suspended into microalgae culture suspensions during the early stages of their growth. Alternatively, CaCO_3_ was precipitated spontaneously by the addition of calcium chloride and sodium carbonate solutions in the culture medium at equimolar, final concentrations of 0.2 M each, pH 8.5. The BET-specific surface area (SSA) of the mechanical mixture of calcite with the AO culture in suspensions, followed by drying at 25 °C was 0.4 m^2^∙g^−1^. The SSA of the solid prepared by the spontaneous precipitation of calcite on air-dried AO culture was 2.2 m^2^∙g^−1^. The morphology of calcite crystallites following the growth of the AO culture did not show significant changes. Small size particles (ca. 50 nm), corresponding to the AO culture cells, were evenly distributed on the flat faces of the rhombohedral calcite crystals (Figure 6a,b).

The crystallites of the solid formed during the precipitation of calcite in suspensions of air-dried AO culture suspended in the supersaturated solutions consisted of hollow vaterite crystallites, confirmed by XRD analysis (Figure 6c,d). The stabilization of the thermodynamically unstable polymorph, vaterite, was possibly due to the presence of soluble organic compounds in the supersaturated solution in which precipitation took place.

**Figure 7:**
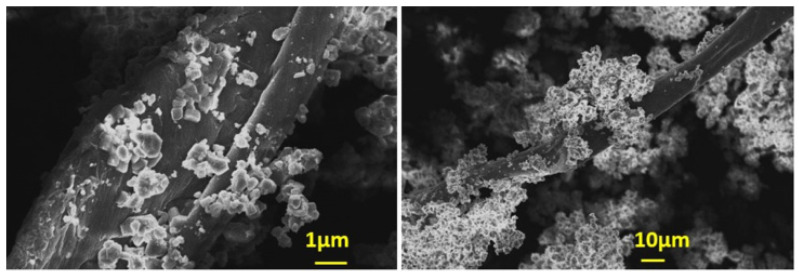
Scanning electron microscope (SEM) photographs of CaCO_3_ deposited in suspensions of dry microalgae.

Examination of the morphology of the composite calcite- AO with SEM (Figure 7) showed that CaCO_3_, precipitating in the suspension of the air-dried AO culture cells in the supersaturated solution, revealed considerable affinity between the organic-inorganic phases. As may be seen in the SEM pictures shown in Figure 7, calcite crystals with the characteristic rhombohedral morphology (shown better on the left picture), grow on chains of AO aggregates.

### 3.2. Spontaneous Precipitation of CaCO_3_ in Supersaturated Solutions: The Metastable Zone Width

#### 3.2.1. The Effect of the Presence of AO Culture Cells in the Supersaturated Solutions

Since the ATR-FTIR study of the dried AO cultures revealed the presence of lipids, it is possible that the presence of AO cultures in supersaturated solutions of CaCO_3_ may favor the selective overgrowth of CaCO_3_, through interactions that may increase the active growth sites in.

Solution supersaturation is the thermodynamic driving force for the precipitation of CaCO_3_ polymorphs from supersaturated solutions. The supersaturation ratio (SR), or saturation index (SI) is defined as the ratio of the ion activity product for the salt considered over the respective thermodynamic solubility product. For the CaCO_3_ polymorphs (vaterite, aragonite, calcite in the order of increasing thermodynamic stability):

(2) $$\mathrm{SR}=\frac{\left({\mathrm{Ca}}^{2+}\right)\left({\mathrm{CO}}_{3}^{2-}\right)}{K_{S,X}^{O}}$$

In Equation (2) ( ) denotes activities of the enclosed ions and $K_{S,X}^{O}$ is the thermodynamic solubility product of polymorph x. The relative supersaturation with respect to polymorph x, σ_x_, is defined as

(3) $$\sigma_{x}={\mathrm{SR}}^{1/2}-1$$

Ion activities in the supersaturated solutions were calculated taking into consideration all equilibria involved, using equilibrium calculations in aqueous systems software, PHREEQC [26].

The supersaturated solutions were prepared in two different ways: (a) mixing equimolar calcium chloride and sodium carbonate solutions, followed by the inoculation with nutrient medium (20 mL) and microalgae culture (5–20 mL), and (b) as in (a) with the difference that the solutions contained sufficient nutrients to ensure the growth of AO cultures.

##### Precipitation of CaCO_3_ in the Presence of Different AO Culture Mass

**Figure 8:**
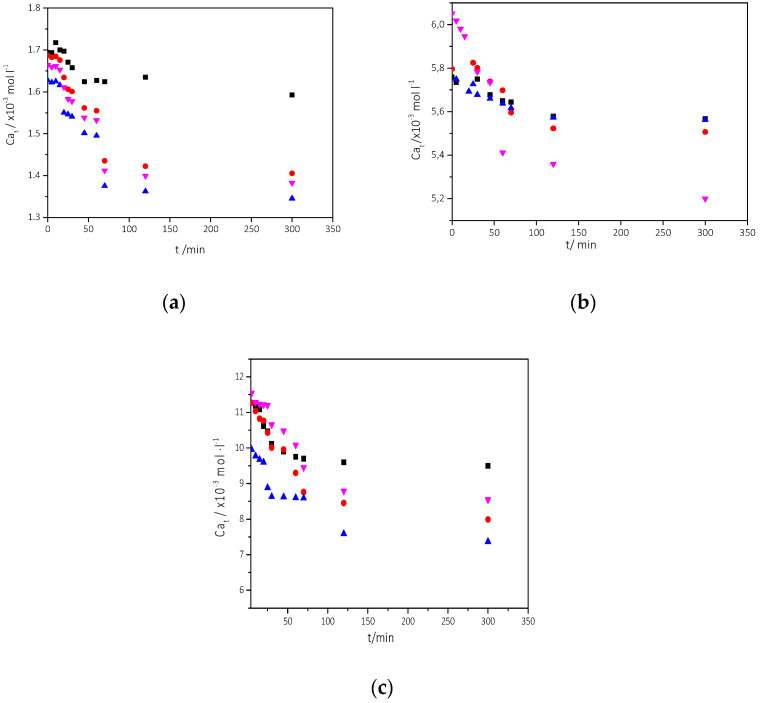
Calcium concentration as a function of time during CaCO_3_ precipitation in the presence of 20 mL nutrient medium and microalgae. (**a**) σ_calcite_ = 0.68, (**b**) σ_calcite_ = 4.43, (**c**) σ_calcite_ = 9.23 (■) Absence of microalgae and nutrient (●) presence of nutrient and 5 mL algae (▲) presence of nutrient and 10 mL algae and (▼) presence of nutrient and 20 mL of algae; 25 °C, pH 8.5, NaCl 0.15 M.

Solutions, supersaturated with respect to all CaCO_3_ polymorphs, with a volume totaling 0.100 dm^3^ were prepared by mixing equal volumes of calcium chloride, sodium bicarbonate, and sodium chloride solutions directly in the reactor, under constant magnetic stirring to ensure homogeneity of the solutions. 20 mL of nutrient medium was added, and the solution pH was adjusted again to 8.5. Next, a quantity of AO culture suspension in the stationary phase of growth was introduced into the stirred solution. The quantities of AO cultures that inoculated the supersaturated solutions were 5, 10, and 20 mL and corresponding to cell numbers 1.26 × 10^−6^, 2.52 × 10^−6^, and 5.04 × 10^−6^ cells, respectively. Following inoculation with AO culture, the solution pH was re-adjusted to pH 8.5 as needed. Samples were withdrawn and filtered through membrane filters then the filtrates were analyzed for calcium. The onset of precipitation was identified from concentration changes from the initial values, and it was free drift. The precipitation of CaCO_3_ lasted for 24 h when it reached a steady state, which was considered to be the completion of precipitation. In this case, the calculation of initial rates is tricky, because the data collected correspond to different supersaturations, i.e., at different conditions which are often influenced by the transformation of less stable intermediate polymorphs to the most stable calcite. It was therefore preferred to calculate the integral rates of CaCO_3_ precipitation from the calcium-time profiles (Figure 8). The experimental conditions are summarized in Table 2.

The concentration of the nutrient medium in the supersaturated solutions was sufficiently low and did not affect the calculated supersaturation with respect to the CaCO_3_ polymorphs. It was also assumed that inside the reactor there was no growth of AO (stationary phase). This assumption was validated from measurements of the concentration of AO cells in the reactor, as may be seen from the data summarized in Table 2. As may be seen from the calcium-time profiles shown in Figure 8, the higher the supersaturation, the higher the change of total calcium (Ca_t_) over the same time period, in agreement with the calculated integral rates, R_a_:

(4) $$R_{a}=\frac{{\left[{\mathrm{Ca}}_{t}\right]}_{0}-{\left[{\mathrm{Ca}}_{t}\right]}_{t}}{∆t}$$

In Equation (4) [Ca_t_]_0_ and [Ca_t_]_t_ are the total calcium concentrations at time t = 0, before the onset of precipitation, and at time t respectively. The rates were calculated over the time period of 100 min (Δt), when calcium concentration attained a plateau value, suggesting the completion of the precipitation process. As may be seen, in Figure 8 in all cases, and over the range of the relative supersaturation values investigated, the precipitation of CaCO_3_ took place practically without any induction time, regardless of the presence of AO culture. It is interesting to note that inoculation of the supersaturated solutions with calcite crystal, was preceded by induction time (ca. 30 min, Figure 8a,b) which may be attributed to the incomplete dispersion of the crystals. In these solutions the precipitation of CaCO_3_ was spontaneous. The presence of AO culture, however, as may be seen, enhanced the precipitation of CaCO_3_ at all supersaturations in the range investigated. Both the rates of precipitation and the total amount of CaCO_3_ precipitated were higher in the presence of AO cultures with the respective nutrient components from the culture medium. It is interesting to note that the rate of precipitation and the total amount of CaCO_3_ precipitated increased with increasing AO culture concentration in the supersaturated solutions, suggesting a direct dependence of the rate of precipitation on the number of active sites present in the AO culture.

**Figure 9:**
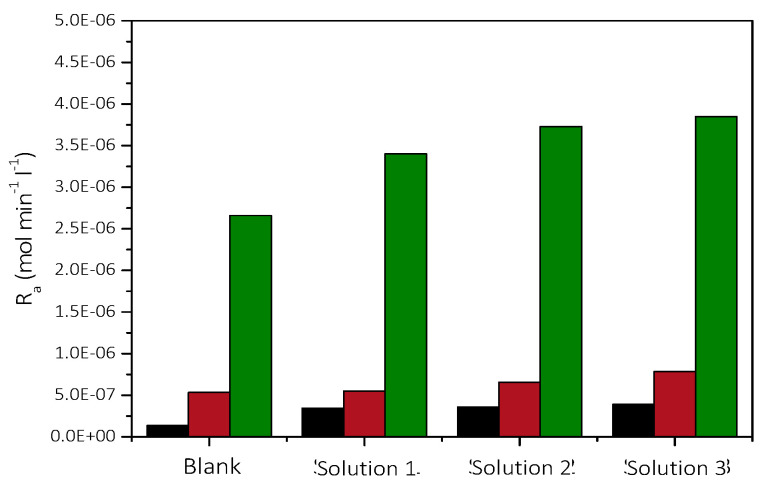
Rates of CaCO_3_ precipitation in the presence of 20 mL nutrient medium and microalgae. Solution 1: 5 mL of microalgae, Solution 2: 10 mL of microalgae, Solution 3: 20 mL of microalgae; 25 °C, pH 8.5, NaCl 0.15 M; AO cells concentration: (ν) 1.26 × 10^−6^ cells·L^−1^; (ν) 2.52 × 10^−6^ cells·L^−1^; (ν) 5.04 × 10^−6^ cells·L^−1^.

As may be seen, the precipitation rate of CaCO_3_ was higher when the concentration of microalgae was higher, suggesting that the nucleation and crystal growth of CaCO_3_ forming is directly related to the number of AO cells which induce the formation of the mineral. the rate of precipitation increased with increasing supersaturation and the available number of AO cells, as may be seen in Figure 9.

**Figure 10:**
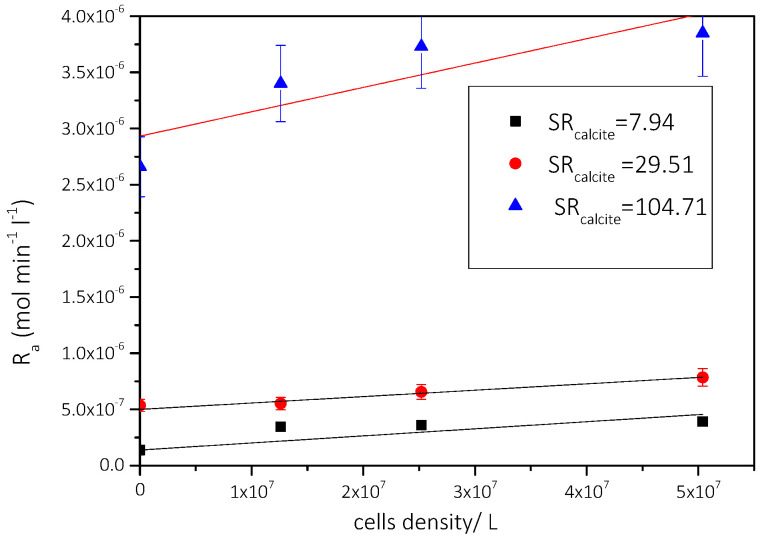
Dependence of the rate of precipitation of CaCO_3_ on the cell concentration of AO culture, in the presence of nutrient medium (20 mL/100 mL of supersaturated solution); 25 °C, pH 8.5, NaCl 0.15 M.

As may be seen in Figure 10, the dependence of the rate of CaCO_3_ precipitation for each of the three supersaturation ratio values studied, on the concentration of AO culture cells in the supersaturated solutions is linear. At the least value of the supersaturations investigated, the rate of precipitation was proportional to the concentration of the AO culture cells in the supersaturated solutions. It is therefore suggested that the AO culture cells provide the active growth sites for the growth of calcite on AO cells and that there is selectivity of calcite growth on the specific type of microalgae cells, mainly consisting of lipids.

The precipitated solid was identified as calcite by XRD (Figure S1, Supplementary Material).

#### 3.2.2. Precipitation of CaCO_3_ in the Presence of AO Culture, Illumination, and Nutrient at Constant Supersaturation

Measurements of rates of precipitation and of the related induction times were measured by the constant supersaturation method [27], which allowed for the accurate measurement of both the induction time and of the rates of precipitation. The presence of nutrients in the AO culture medium is necessary for the growth of the respective cells, we have tested the use of modified (lower concentrations of the metal ion components) nutrient medium (Table 1). This was necessary, to avoid precipitation of metal carbonates other than CaCO_3_ (MgCO_3_ or FeCO_3_ e.g.,). In this case, larger volume of the supersaturated solutions and a higher capacity reactor (0.5 dm^3^) were used. The supersaturated solutions were prepared in the reactor, as already described, at 25 ± 0.5 °C by mixing equimolar solutions of calcium and carbonate (0.2 dm^3^ each), with the simultaneous addition of precisely measured amounts of nutrients (Table 1). The supersaturated solutions with the nutrients were adjusted to pH 8.5 with standard NaOH solution. The solutions were homogenized by magnetic stirring through the precipitation process. 5 mL of the AO culture was injected into the supersaturated solutions. The kinetics of precipitation of CaCO_3_ was monitored both in the absence (control) and in the presence of nutrient medium and illumination.

**Figure 11:**
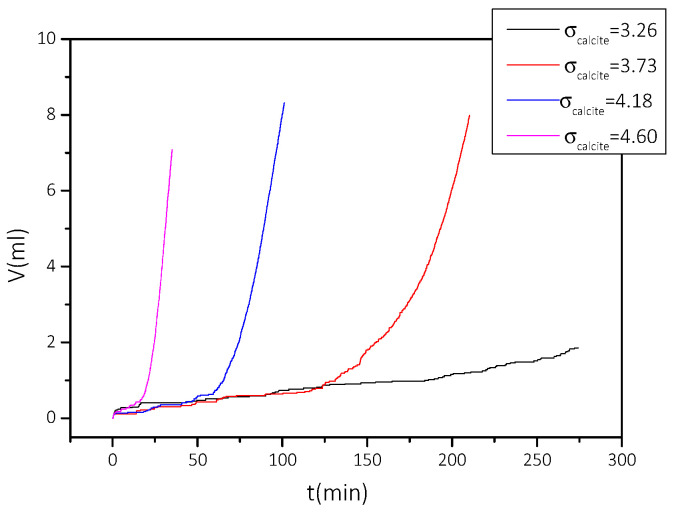
Titrant solution volume added to the reactor to maintain supersaturation, in the presence of medium and 5 mL of algae under illumination, as a function of time: 25 °C, pH 8.5, NaCl 0.15 M.

From the volume of titrant solutions added (Figure 11), to maintain constant supersaturation as a function of time, the precipitation rates were calculated according to Equation (4). The measured rates of precipitation of CaCO_3_ and the respective solution conditions are summarized in Table 3.

**Figure 12:**
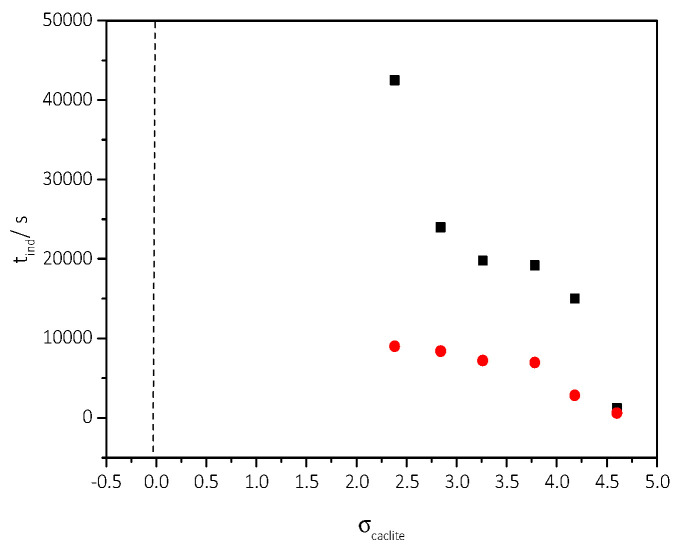
Stability diagrams of CaCO_3_ supersaturated solutions, in the presence of medium and 5 mL AO culture slurry, under illumination. Induction time as a function of relative supersaturation. (■) in the absence of AO culture; (●) in the presence of AO culture; 25 °C, pH 8.5, ΝaCl 0.15 M. Measurements at constant supersaturation.

From the data in Table 3, the stability diagram of the supersaturated with respect to calcite solutions was drawn, in the presence of 5 mL of AO culture, nutrient medium, and illumination. The stability diagram is shown in Figure 12. In the same graph, the stability of the supersaturated solutions in the absence of AO culture, is also shown. As may be seen, the presence of the AO culture resulted in a narrower stability zone of the supersaturated CaCO_3_ solutions.

**Figure 13:**
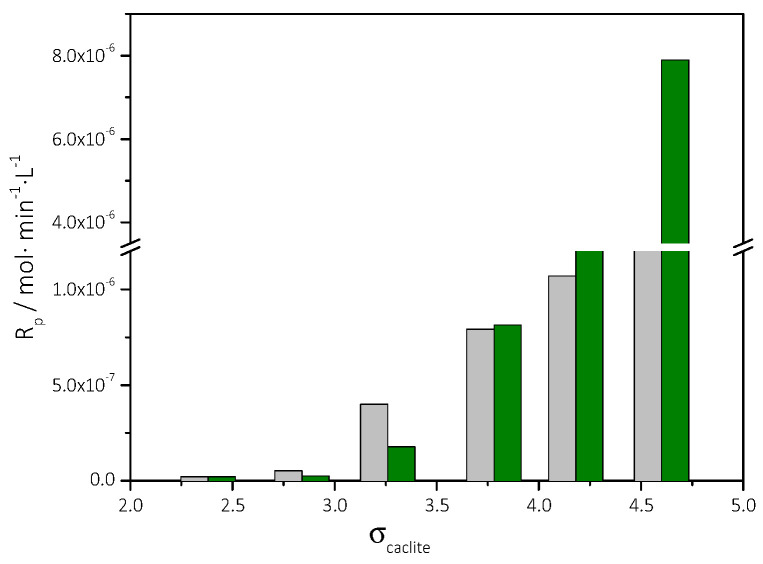
Precipitation of CaCO_3_ in supersaturated solutions at constant supersaturation. Rates of precipitation of CaCO_3_, as a function of the relative supersaturation with respect to calcite. In the absence (gray bars) and in the presence of AO culture medium and 5 mL of AO culture; supersaturated solutions with AO and culture medium with illumination (green bars); 25 °C, pH 8.5, NaCl 0.15 M.

Up to a relative supersaturation value of about 3.8, there was no significant difference between the rate of CaCO_3_ precipitation in the presence or in the absence of AO culture. For relative supersaturation values exceeding 4.3 the rate of CaCO_3_ precipitation increased significantly in the presence of AO culture by up to 100% in comparison with the reference value (Figure 13). It may be suggested that the interaction between the free calcium ions with the lipids rich AO cells resulted in the creation of additional active growth sites. Consequently, it may be suggested that locally higher supersaturation was developed on the surface of the AO cells in comparison with the respective in the bulk.

**Figure 14:**
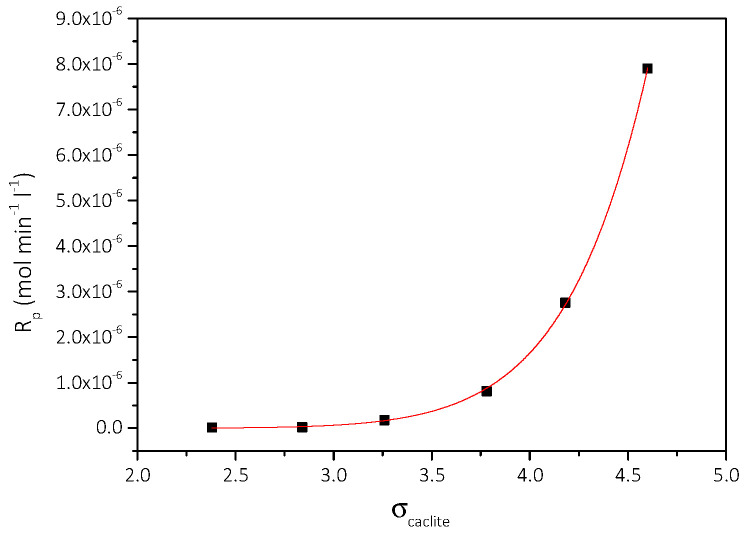
Precipitation of CaCO_3_ in supersaturated solutions at constant supersaturation, in the presence of culture medium and 5 mL of AO culture, under illumination. Precipitation rate as a function of relative supersaturation; pH 8.5, 25 °C, NaCl 0.15 M.

The dependence of the rate of calcite precipitation, R_p_, as a function of corresponding relative supersaturation, σ_calcite_, is shown in Figure 14. Fitting of the data to the semiempirical power law:

(5) $$R_{p}=k_{p}\sigma_{\mathrm{calcite}}^{n}$$

where k_p_ and n are the apparent constant and the apparent order of the precipitation, respectively. K_p_ is a function of the active sites for crystal growth and n is indicative of the mechanism of precipitation. Fitting of the data in Equation (5) suggested that n = 11. The meaning of the high value for the apparent order is that the mechanism of CaCO_3_ precipitation is described according to the polynuclear model, according to which several nuclei are formed and grow simultaneously on the substrate. The data were fitted to respective rate expression for the polynuclear model [28]:

(6) $$R_{p}=\mathrm{Cf}\left({\mathrm{SR}}_{\mathrm{calcite}}\right)\exp\left(-\frac{{\beta \gamma }_{s\;}^{2}V_{m}^{4/3}}{3{\left(\mathrm{kT}\right)}^{2}{\mathrm{lnSR}}_{\mathrm{calcite}}}\right)$$

In Equation (6) C is a constant, f(S) is a function of the saturation ratio:

(7) $$f\left({\mathrm{SR}}_{\mathrm{calcite}}\right)={\mathrm{SR}}_{\mathrm{calcite}}^{7/6}{\left({\mathrm{SR}}_{\mathrm{calcite}}-1\right)}^{2/3}\ln{\left({\mathrm{SR}}_{\mathrm{calcite}}\right)}^{1/6}$$

β is a shape factor (b = 1 in this case), γ_s_ the surface energy of the nuclei forming at the active growth sites, V_m_, the molecular volume of the solid growing, k is Boltzmann’ s constant and T the absolute temperature.

The plot of $\ln\left(\frac{R_{p}}{f\left({\mathrm{SR}}_{\mathrm{calcite}}\right)}\right)$ as a function of $1/\ln\left({\mathrm{SR}}_{\mathrm{calcite}}\right)$ is shown in the plot of Figure 13. The excellent fit suggested than in the presence of AO cultures the growth of calcite is described satisfactorily with the polynuclear model.

**Figure 15:**
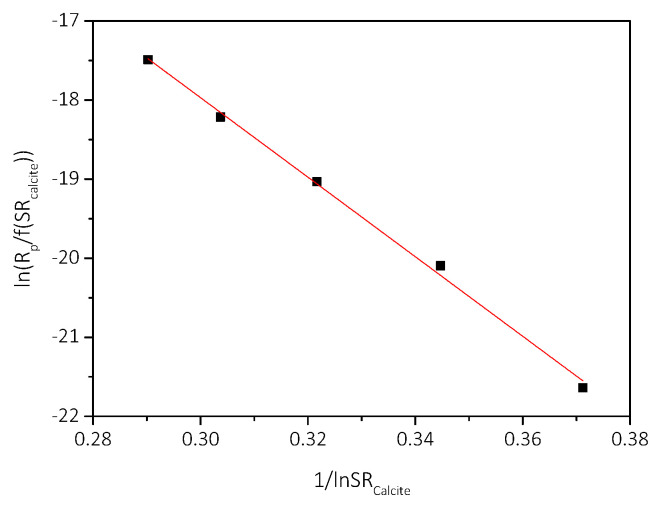
Precipitation of CaCO_3_ in supersaturated solutions at constant supersaturation, in the presence of medium and 5 mL of AO culture, under illumination. Plot of the precipitation rates as a function of relative supersaturation, according to the polynuclear model; pH 8.5, 25 °C, NaCl 0.15 M.

From the slope of the linear fit according to Equation (6), shown in Figure 15, the surface energy of calcite was calculated equal to 56 mJ∙m^−2^.

### 3.3. Precipitation of CaCO_3_ in Supersaturated Solutions Inoculated with AO Cultures

The selectivity of AO microalgae to induce crystal growth of CaCO_3_ was examined in supersaturated solutions, using the free drift method, described in Section 2.2.1. From the results presented so far, it may be suggested that photosynthesis is important for the precipitation of CaCO_3_. Therefore, the role of photosynthesis needs further investigation. The formation of CaCO_3_ was induced by the inoculation of the supersaturated solutions with substrates that possess the active sites needed for the nucleation and subsequent growth of an insoluble salt. For example, the inoculation of supersaturated CaCO_3_ solutions with calcite seed crystals is known to induce crystal growth of calcite without nucleation [29]. When the inoculation solids are different than calcite, the existence of a nucleation barrier is anticipated. Four different substrates were used to inoculate the supersaturated CaCO_3_ solutions: (i) AO microalgae, dried at 70 °C for 24 h (SSA < 0.1 m^2^g^−1^), (ii) AO microalgae, air dried at 25 °C for 2 days (SSA = 2.64 m^2^·g^−1^), (iii) AO culture grown on calcite crystals dried at 25 °C for 2 days (SSA = 0.38 m^2^·g^−1^) and (iv) calcite crystals precipitated on dry AO microalgae, dried at 25 °C (SSA = 2.2 m^2^·g^−1^).

The study was done in the range of relative supersaturation with respect to calcite, 2.38–4.6 (corresponding to Total calcium, Ca_t_ = Total Carbonate, C_t_ range 3.5–6 mM) for ionic strength 0.15 M NaCl. Following the inoculation of the supersaturated solutions with the study substrates, the pH value was monitored.

Inoculation of the supersaturated solutions with AO culture dried at 70 °C for 24 h, did not induce precipitation of CaCO_3_ over the range of supersaturations studied as shown by the stability of pH of the solutions and the calcium concentration in the supersaturated solutions. The lack of precipitation may be due to the denaturation of proteins and reduction of the active sites, which may be ionized functional groups on the cell membranes [30].

**Figure 16:**
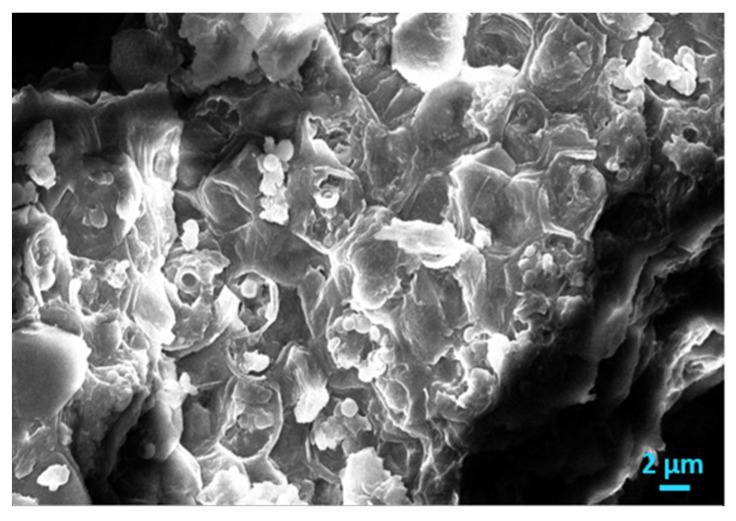
Scanning electron microscope (SEM) photos of AO culture cells, dried at 70 °C.

Dried AO culture, which is not able to carry out photosynthesis producing chlorophyll was used to inoculate supersaturated solutions, to test its ability to induce nucleation of CaCO_3_. At temperatures exceeding 40–45 °C, the rate of photosynthesis decreases rapidly [31]. Apparently, the activity of the cells was canceled because of the change in the conformation of protein and lipid molecules present in the cell membranes, which are responsible for inducing nucleation and growth of CaCO_3_. As may be seen from the examination of the morphology with SEM, presented in Figure 16, the inactivation of the microalgae could possibly be due to the lysis of its cells associated with enzymatic hydrolysis. The destruction of the cell membranes as well as the components of the extracellular polymeric membrane, most likely contribute to the precipitation of CaCO_3_. It has been shown that membrane destruction accompanied by structural alterations, may also be caused by the exposure of microalgae to ultrasound or under conditions of intense homogenization [32].

In the range of supersaturation values with respect to calcite, 2.38 < σ_calcite_ < 4.6, the solutions, were inoculated with 100 mg of air-dried AO (BET SSA 2.64 m^2^·g^−1^) (25 °C). The precipitation was monitored for 24 h.

**Figure 17:**
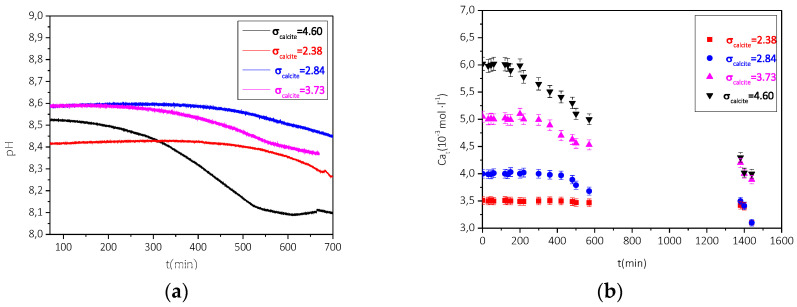
Precipitation of CaCO_3_ in supersaturated solutions inoculated with 100 mg air dried AO. (**a**) Change of pH as a function of time. Initial pH 8.5, 25 °C, 0.15 M NaCl & (**b**) Calcium (total), Ca_t_ as a function of time pH 8.5, 25 °C, 0.15 M NaCl.

The profiles of the pH and total calcium, Ca_t_ are shown in Figure 17a,b.

As may be seen from the pH- time and Ca_t_- time profile the precipitation was preceded by induction times inversely proportional to the relative supersaturation of the solutions with respect to calcite. The rates of precipitation were calculated from the Ca_t_-time profiles at t→0 (initial rates). The conditions at which CaCO_3_ precipitated following inoculation with air-dried AO cells, the measured induction times, and the rates of precipitation calculated are summarized in Table 4.

From the XRD patterns obtained, it was found that the solid formed was calcite and vaterite for relative supersaturation values higher than 3.73 (Supplementary Material Figure S1).

It should be noted that the formation of amorphous CaCO_3_ (ACC) was identified, suggesting that at σ_calcite_ = 2.38, this unstable phase was stabilized as verified by XRD and SEM (Supplementary Material Figure S2). The formation of ACC mediated by microorganisms has been reported both in nature [33] and in laboratory studies [34]. It should be noted that ACC was converted into vaterite within the first hour or two and completely to calcite past 24 h of contact of the solid with the mother supersaturated liquor.

Next, the inoculation of the supersaturated solutions with AO culture developed on calcite crystals was investigated. The solid (BET SSA 0.38 m^2^·g^−1^) was air-dried past 24 h of culture development and was used to inoculate supersaturated CaCO_3_ solutions, 2.38 < σ_calcite_ < 4.60. The precipitation of CaCO_3_ in the respective supersaturated solutions lasted for 24 h.

In this case, the substrate used to inoculate the supersaturated solutions failed to induce the precipitation of CaCO_3_.

As already mentioned, (Section 3.2.1), the precipitation of CaCO_3_ takes place in air-dried AO culture. The mixed calcite-air-dried AO culture solid (BET SSA 2.2 m^2^·g^−1^) was used to inoculate supersaturated solutions (2.38 < σ_calcite_ < 4.60). The precipitation of CaCO_3_ was monitored from pH and total calcium concentration as a function of time (free drift). The experimental conditions measured induction times preceding precipitation, and the rates (initial) of CaCO_3_ precipitation are summarized in Table 5.

**Figure 18:**
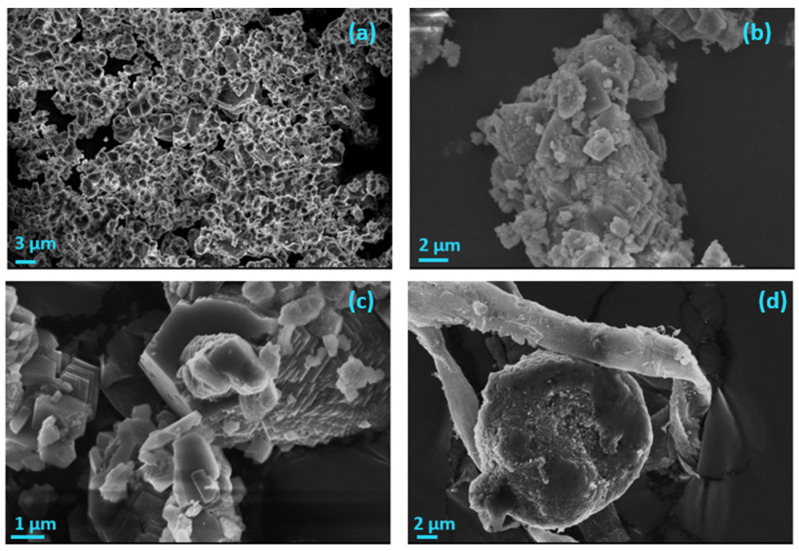
SEM photographs of the precipitate in stable supersaturated solutions of CaCO_3_, σ_calcite_ = 3.73, on mixed calcite-air dried AO cells; (**a**) calcite crystallites aggregate; (**b**) calcite rhombohedral crystal aggregates; (**c**) calcite crystals and aggregates; (**d**) calcite crystallite associated with dry AO cells showing association with cell membrane; pH 8.5, 0.15 M NaCl, 25 °C.

The precipitate consisted of a mixture of vaterite and calcite The morphology of the precipitate showed CaCO_3_ aggregates, (Figure 18a), in which the calcite rhombohedra (Figure 18b,c), were associated with AO cell remnants (Figure 18d).

**Figure 19:**
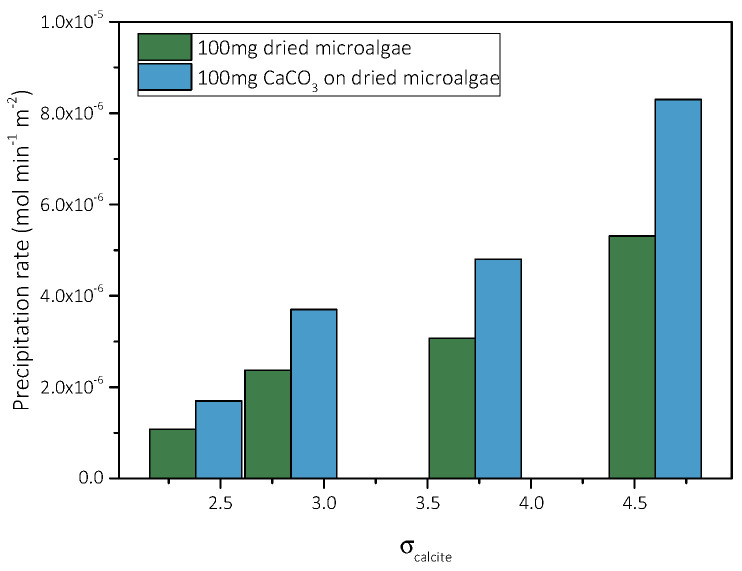
Rate of precipitation of CaCO_3_ in stable supersaturated solutions inoculated with air-dried AO culture (green bars) and with mixed calcite precipitated in air dried AO culture (blue bars), as a function of supersaturation; Initial pH 8.5, 0.15 M NaCl, 25 °C.

Comparison of the rates of precipitation of CaCO_3_ with the corresponding solutions of the same supersaturation were seeded with AO culture dried at 25 °C (air dried) and showed accelerated precipitation upon inoculation with the mixed AO dried culture with CaCO_3_ crystals, as may be seen in Figure 19.

## 4. Conclusions

*Acutodesmus Obliquus* (AO) cultures were grown under periodic illumination (light:dark = 16:8), in the aqueous nutrient medium Basal Bold’s Medium, at pH 6.5 and temperature 25 °C. The growth of AO was monitored from measurements of the produced chlorophyll. The ability of AO cultures to induce selectively the precipitation of calcium carbonate (CaCO_3_) from stable CaCO_3_ supersaturated solutions was tested by monitoring the free-drift precipitation in supersaturated solutions. For AO obtained in suspension form, the number of cells was very important. The rates of precipitation of CaCO_3_ increased linearly with AO culture cells as a function of time. The rates of CaCO_3_ precipitation in the presence of AO and the respective nutrients of AO increased by up to 79% when the supersaturated solutions were illuminated, most likely because of the increase in AO cell concentration and contribution from the CO_2_ production from the photosynthesis activity. Air dried (25 °C) AO culture induced the precipitation of CaCO_3_ from stable supersaturated solutions because the cells and cell membranes provided the necessary active sites for the nucleation and crystal growth of CaCO_3_. On the contrary, AO culture cells dried at 70 °C, did not show any activity with respect to the selective growth of CaCO_3_, because of the denaturation of the cell membrane components (proteins-lipids). It is interesting to note, that CaCO_3_ precipitated because of the presence of air-dried AO cells stabilized amorphous CaCO_3_ (ACC) which with time was converted to more stable CaCO_3_, such as vaterite and calcite. The latter was the only CaCO_3_ polymorph identified past contact with the mother supersaturated aqueous solution for 24 h.

## Figures and Tables

**Table 1:** Composition of modified AO culture medium suitable * for the supersaturated CaCO_3_ solutions.

Component	Concentration/mM
MgSO_4_∙7H_2_O	0.400
KNO_3_	0.247
NaNO_2_	0.005
H_3_BO_3_	0.040
FeCl_3_∙2H_2_O	0.006
MgSO_4_∙7H_2_O	0.400

* Modification from the recommended optimal concentrations for the AO culture so that the supersaturated solutions were stable and did not result in the precipitation of magnesium or iron carbonates.

**Table 2:** Free drift CaCO_3_ precipitation in the presence of 20 mL nutrient medium and AO culture. Initial total calcium, Cat, concentrations, calculated supersaturation ratio, SR, values with respect to calcite, relative supersaturation with respect to calcite, AO cell concentration and rates of CaCO_3_ precipitation (integral rates)*;* 25 °C, pH 8.5, NaCl 0.15 M.

Solution	Ca_t_/×10^−3^ mol∙L^−1^	Supersaturation Ratio, SR_calcite_	Relative Supersaturation σ_calcite_	AO Cell Concentration/×10^7^ cells·L^−1^	Precipitation Rate, R_p_/×10^−7^ mol min^−1^ L^−1^
1	1.7	7.94	0.68	1.26	1.4
				2.52	3.5
				5.04	3.6
2	5.8	29.51	4.43	1.26	5.4
				2.52	5.5
				5.04	6.6
3	11.6	104.71	9.23	1.26	26.6
				2.52	34.0
				5.04	37.3

**Table 3:** Precipitation of CaCO_3_ in supersaturated solutions at constant supersaturation, in the presence of culture medium and 5 mL of AO culture, under illumination. Calcium (total) concentration in the supersaturated solutions, relative supersaturation with respect to calcite, induction time preceding the onset of precipitation, and the precipitation rate of CaCO_3_; 25 °C, pH 8.5, NaCl 0.15 M.

Ca_t_/×10^−3^ mol∙L^−1^	Relative Supersaturation, σ_calcite_	Induction Time, t_ind/_min	Precipitation Rate, R_p_/×10^−7^ mol∙min^−1^∙L^−1^
3.5	2.38	150	0.21
4.0	2.84	140	0.24
4.5	3.26	120	1.78
5.0	3.73	116	8.15
5.5	4.18	47	27.5
6.0	4.60	10	79.0

**Table 4:** Precipitation of CaCO_3_ in stable supersaturated solutions, inoculated with, 100 mg of air-dried AO. Concentration (total) of calcium and total carbonate relative supersaturation with respect to calcite, induction times measured, and rates of CaCO_3_ precipitation; pH 8.5, 25 °C, 0.15 M NaCl.

Total Calcium, Ca_t_ = Total Carbonate, C_t_/×10^−3^ mol∙L^−1^	Relative Supersaturation, σ_calcite_	Induction Time, t_ind/_min	Precipitation Rate (t→0)/×10^−6^ mol∙min^−1^∙L^−1^∙m^−2^
3.5	2.38	463	1.1
4.0	2.84	406	2.4
5.0	3.73	359	3.1
6.0	4.60	272	5.3

**Table 5:** Precipitation of CaCO_3_, in stable supersaturated solutions, inoculated with 100 mg of calcite-AO culture powdered solid; Total Calcium concentration Ca_t_ (=C_t_, total carbonate concentration), relative supersaturation with respect to calcite, induction time and precipitation rate; Initial pH 8.5, 0.15 M NaCl, 25 °C.

Total Calcium, Ca_t_ = Total Carbonate, C_t_/×10^−3^ mol∙L^−1^	Relative Supersaturation, σ_calcite_	Induction Time, t_ind/_min	Precipitation Rate (t→0)/×10^−6^ mol∙min^−1^∙L^−1^∙m^−2^
3.5	2.38	435	1.7
4.0	2.84	385	3.7
5.0	3.73	242	4.8
6.0	4.60	208	8.3

## Data Availability

Not applicable.

## Supplementary Materials

The following supporting information can be downloaded at: https://www.mdpi.com/article/10.3390/biomimetics7040140/s1, Figure S1: Precipitation (free drift) of calcium carbonate in supersaturated solutions (SR_calcite_ = 11.48), at constant temperature, 25 °C, in the presence of 100 mg of AO culture, dried in ambient air. X-ray diffraction pattern of the precipitated solid; Figure S2: SEM photo of calcium carbonate precipitated in supersaturated solutions (SR_calcite_ = 11.48), at a constant temperature of 25 °C with free change of concentrations, in the presence of 100 mg of dry microalgae, dried in ambient air at 25 °C.
