# Supplementary material for: Calcium Carbonate Mineralization of Microalgae
Source: Biomimetics (Basel). 2022 Sep 23;7(4):140. doi: 10.3390/biomimetics7040140 (PMC9589979; doi:10.3390/biomimetics7040140)
Supplement: Supplementary file 1 [file biomimetics-07-00140-s001.zip › biomimetics-1915283-supplementary.pdf]

**Supplementary Materials:**

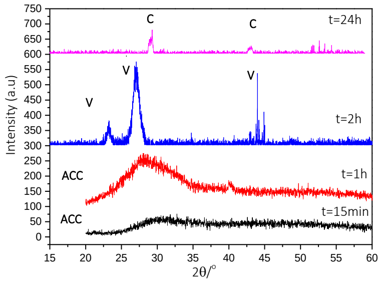


**Figure S1.** Precipitation (free drift) of calcium carbonate in supersaturated solutions ($SR_{calcite}$ =11.48), at constant temperature, 25°C, in the presence of 100mg of AO culture, dried in ambient air. X-ray diffraction pattern of the precipitated solid.

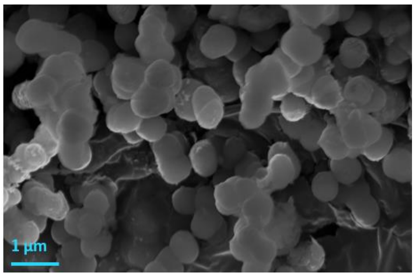


**Figure S2.** SEM photo of calcium carbonate precipitated in supersaturated solutions ($SR_{calcite}$=11.48), at a constant temperature of 25°C with free change of concentrations, in the presence of 100mg of dry microalgae, dried in ambient air at 25°C.
